# Cannabinoid receptor 2 deficiency exacerbates inflammation and neutrophil recruitment

**DOI:** 10.1096/fj.201802524R

**Published:** 2019-02-25

**Authors:** Theodore S. Kapellos, Lewis Taylor, Alexander Feuerborn, Sophia Valaris, Mohammed T. Hussain, G. E. Rainger, David R. Greaves, Asif J. Iqbal

**Affiliations:** * Sir William Dunn School of Pathology, University of Oxford, Oxford, United Kingdom;; † Institute of Cardiovascular Sciences, College of Medical and Dental Sciences, University of Birmingham, Birmingham, United Kingdom

**Keywords:** innate immunity, leukocyte trafficking, adhesion

## Abstract

Cannabinoid receptor (CB)_2_ is an immune cell–localized GPCR that has been hypothesized to regulate the magnitude of inflammatory responses. However, there is currently no consensus as to the mechanism by which CB_2_ mediates its anti-inflammatory effects *in vivo*. To address this question, we employed a murine dorsal air pouch model with wild-type and CB_2_^−/−^ 8–12-wk-old female and male C57BL/6 mice and found that acute neutrophil and lymphocyte antigen 6 complex, locus C^hi^ monocyte recruitment in response to Zymosan was significantly enhanced in CB_2_^−/−^ mice. Additionally, levels of matrix metalloproteinase 9 and the chemokines C-C motif chemokine ligand (CCL)2, CCL4, and C-X-C motif chemokine ligand 10 in CB_2_^−/−^ pouch exudates were elevated at earlier time points. Importantly, using mixed bone marrow chimeras, we revealed that the proinflammatory phenotype in CB_2_^−/−^ mice is neutrophil-intrinsic rather than stromal cell–dependent. Indeed, neutrophils isolated from CB_2_^−/−^ mice exhibited an enhanced migration-related transcriptional profile and increased adhesive phenotype, and treatment of human neutrophils with a CB_2_ agonist blocked their endothelial transmigration. Overall, we have demonstrated that CB_2_ plays a nonredundant role during acute neutrophil mobilization to sites of inflammation and, as such, it could represent a therapeutic target for the development of novel anti-inflammatory compounds to treat inflammatory human diseases.—Kapellos, T. S., Taylor, L., Feuerborn, A., Valaris, S., Hussain, M. T., Rainger, G. E., Greaves, D. R., Iqbal, A. J. Cannabinoid receptor 2 deficiency exacerbates inflammation and neutrophil recruitment.

Since the discovery of cannabinoid receptor (CB) 1, CB_2_, and their endogenous lipid ligands (known as the endocannabinoids), almost 3 decades of work has now established that these 2 GPCRs and their cognate ligands, alongside the enzymes that synthesize and degrade these endogenous lipids, make up the endocannabinoid system (1–4).

Both CB_1_ and CB_2_ are class A rhodopsin-like GPCRs and are G_i/o_-coupled; therefore, their ligation results in the inhibition of adenylyl cyclase and the lowering of intracellular cAMP levels (5). However, signaling *via* the G-protein βγ subunits can also lead to intracellular Ca^2+^ release, activation of ion channels, β-arrestin recruitment, and PI3K and mitogen-activated protein signaling (6, 7).

CB_1_ and CB_2_ exhibit distinct expression patterns, with CB_1_ highly enriched throughout the CNS (8) and CB_2_ predominantly expressed in leukocytes, such as B cells, NK cells, mast cells, neutrophils, monocytes, and T cells (6). However, recent data have demonstrated that CB_2_ expression can also be found in the brain (9). The presence of CB_2_ on immune cells has resulted in focused academic interest surrounding the role CB_2_ plays in inflammation and inflammatory disease, and indeed, CB_2_ ligation has been demonstrated to regulate various aspects of immune cell function [reviewed in Turcotte *et al.* (10)]. For instance, it was shown that CB_2_ promotes the retention of B cells in the murine spleen (11) and it plays an important role in the control of acute inflammatory responses (12, 13).

However, and despite the fact that CB_2_ activation has been found to have a positive outcome in a range of acute and chronic inflammatory animal models of diseases, such as inflammatory bowel disease (14, 15), sepsis (16, 17), multiple sclerosis (18, 19), ischemia reperfusion injury (20–23), and atherosclerosis (24, 25), the exact mechanism underpinning these beneficial effects remains unknown. One hypothesis put forward is that activation of CB_2_ blocks immune cell chemotaxis; however, we recently found that CB_2_ does not play a role in regulating primary macrophage chemotaxis (26). Additionally, most previous studies investigating CB_2_ within inflammation have used indirect or semiquantitative measures of immune cell recruitment and only examine a single time point.

To overcome these limitations, we conducted a fully quantitative analysis of the effect of global genetic deletion of CB_2_ on neutrophil, and other innate immune cell, recruitment in a model of self-resolving acute inflammation at multiple time points. We report that CB_2_ suppresses neutrophil recruitment to the dorsal air pouch *via* a neutrophil-intrinsic mechanism. Neutrophils of CB_2_-deficient animals have a dysregulated transcriptomic profile consistent with a promigratory phenotype that is manifest in increased adherence of murine CB_2_^−/−^ neutrophils to intercellular adhesion molecule (ICAM)1 *in vitro* and decreased adhesion and transmigration of CB_2_ agonist–treated human neutrophils to activated endothelial cells.

## MATERIALS AND METHODS

### Reagents

Bio-gel polyacrylamide beads (P-100 fine, 45–90 μm) were purchased from Bio-Rad (Hercules, CA, USA); anti-mouse CD45.1 (A20), CD45.2 (104), CD11b (M1/70), CD115 (AFS98), lymphocyte antigen 6 complex, locus C (Ly-6C) (HK1.4), and lymphocyte antigen 6 complex, locus G (Ly-6G) (1A8) were purchased from BioLegend (San Diego, CA, USA); anti-mouse CD45 (30-F11) was obtained from BD Biosciences (San Jose, CA, USA); quantitative PCR (qPCR) primers were purchased from Qiagen (Germantown, MD, USA); and all cell culture media and reagents were obtained from GE Healthcare (Waukesha, WI, USA).

### Animals

Animal studies were performed with local ethical approval from the Dunn School of Pathology Animal Welfare Ethical Review Board and according to the United Kingdom Home Office regulations (Guidance on the Operation of Animals, Scientific Procedures Act, 1986). C57BL/6 and B6.SJL mice were obtained directly from the Biomedical Sciences Unit (Oxford, United Kingdom) and were housed in a 12-h light/dark cycle with free access to food and water. B6.129P2-Cnr2^tm1Dgen^/J mice (herein referred to as CB_2_^−/−^ mice) were purchased from the The Jackson Laboratory (Bar Harbor, ME, USA). These mice were originally engineered by Deltagen (San Mateo, CA, USA) and were backcrossed onto the C57BL/6 background for at least 10 generations. It should be noted that CB_2_ mRNA was detected in tissues taken from CB_2_^−/−^ animals. However, PCR combined with Sanger sequencing confirmed that this was not the full-length CB_2_ transcript and was therefore unlikely to be translated into functional protein (unpublished results). Female 8–16-wk-old animals (25–30 g) were used in all experiments (unless otherwise specified), and power calculations were carried out in advance to determine the minimum number needed to detect an effect size of at least 30% with *P* < 0.05.

### Dorsal air pouch inflammation model

Female mice were anesthetized, and air pouches were created by dorsal subcutaneous injection of 2.5 ml sterile air on d 0 and 3. On d 6, animals were anesthetized and were injected with 100 μg Zymosan (MilliporeSigma, Burlington, MA, USA) in 500 μl PBS. Pouches were lavaged 2, 6, 16, or 48 h after Zymosan injection with 3 ml PBS containing 2 mM EDTA. Blood was collected into EDTA-coated tubes.

### Flow cytometry

Dorsal air pouch exudates (300 μl) were centrifuged at 200 *g* for 5 min at 4°C. Cell pellets were resuspended in 50 μl fluorescence-activated cell sorting (FACS) buffer [PBS containing 2% fetal calf serum (FCS), 25 mM HEPES, and 5 mM EDTA] and were blocked with 116 μg/ml mouse IgG (Jackson ImmunoResearch, West Grove, PA, USA) and 6.6 μg/ml mouse SeroBlock FcγR (Bio-Rad) for 15 min on ice. Antibody staining was performed for 30 min on ice and protected from light before resuspending cells in 1% paraformaldehyde. All samples were run on a Dako Cyan ADP flow cytometer (Beckman Coulter, Brea, CA, USA) and data were analyzed using FlowJo v.10 software (BD Biosciences). Peripheral blood (50 μl) was mixed 1:1 with blocking solution, and cells were stained as above. Red blood cells were subsequently lysed with 3 ml BD FACS lysing solution (BD Biosciences) for 15 min at room temperature. Cells were washed twice with 1 ml FACS buffer before final resuspension in 1% paraformaldehyde.

### Generation of mixed bone marrow chimeric animals

Bone marrow cells were isolated from female B6.SJL (CD45.1^+^) and CB_2_^−/−^ animals (CD45.2^+^). Red blood cell lysis was carried out in 10 ml of ammonium-chloride-potassium lysis buffer (155 mM NH_4_Cl; 10 mM KHCO_3_; 100 μM EDTA) for 5 min at room temperature before centrifugation at 250 *g* for 5 min at 4°C. Cells were then resuspended in PBS for counting, and equal numbers of CD45.1^+^ and CD45.2^+^ cells were mixed to a final concentration of 2.5 × 10^7^ cells/ml. Female C57BL/6 or CB_2_^−/−^ recipient animals were sublethally irradiated with 2 doses of 5 Gy separated by a 3-h gap. They were then injected intravenously with 200 μl of the bone marrow cell suspension (5 × 10^6^ cells/mouse) and assessed after 5 wk for bone marrow transplantation efficiency by blood flow cytometry. Dorsal air pouches were established 1 wk later.

### Cell counting

CountBright Absolute Counting Beads (50 μl; Thermo Fisher Scientific, Waltham, MA, USA) were added to 300 μl dorsal air pouch exudates, and samples were analyzed by flow cytometry gating separately on beads and cells.

### Cytokine, chemokine, and metalloproteinase level assessment

The concentration of TNF-α, IL-6, matrix metalloproteinase 9 (MMP-9), C-C motif chemokine ligand (CCL) 2, CCL3, CCL4, C-X-C motif chemokine ligand (CXCL) 1, CXCL2, CXCL5, and CXCL10 was determined using DuoSet sandwich ELISA and Magnetic Luminex Screening Assays (R&D Systems, Minneapolis, MN, USA) following the manufacturer’s guidelines. Optical density absorbance was measured at 450 nm with correction at 570 nm using a Pherastar plate reader (BMG Labtech, Offenburg, Germany). Luminescence was measured with a Bio-Plex 200 System (Bio-Rad). The amount of each analyte was interpolated from the protein standard curve and multiplied with the appropriate dilution factor.

### Culture and stimulation of dorsal air pouch fibroblasts

Dorsal air pouches were injected with 3 ml prewarmed TrypLE Express (Thermo Fisher Scientific) for 15 min at 37°C, and exudates were collected into 6 ml DMEM containing 10% FCS. Cells were then passed through a 45-μm cell strainer and centrifuged at 200 *g* for 5 min at 4°C. They were then resuspended in 3 ml DMEM containing 10% FCS and seeded into 6-well plates. Medium was replaced every 2 d until cells reached 90% confluency. Fibroblasts were detached from plastic following a 5-min incubation with TrypLE Express at 37°C and resuspended in DMEM containing 10% FCS for cell counting using trypan blue exclusion. Cells (2 × 10^6^ cells/ml) were plated in 12-well plates overnight at 37°C/5% CO_2_ for stimulation with vehicle or Zymosan (10 μg/ml) for 6 h.

### RNA extraction and cDNA synthesis

RNA extraction was conducted using the RNeasy Mini Kit (Qiagen). RNA concentration and quality were determined using a NanoDrop ND-1000 spectrophotometer (Thermo Fisher Scientific). cDNA was synthesized from purified RNA (500 ng) using the QuantiTect Reverse Transcription Kit (Qiagen) following the manufacturer’s protocol.

### qPCR

Actin γ1 (Actg1), cannabinoid receptor 2 (*Cnr2*), vimentin (*Vim*), *Tlr2* (Toll-like receptor 2), C-type lectin domain family 7, member a (*Clec7a*) (Dectin-1), C-C chemokine receptor 7 (*Ccr7*), *Ccl22,* and *Cxcl10* expression was determined in qPCR experiments. cDNA (50 ng) was used as a template, and master mix reactions contained 2× SYBR Select PCR master mix (Thermo Fisher Scientific), primers (500 nM) (**Table 1**), and nuclease-free water. The thermal profile used consisted of a denaturation step at 95°C for 5 min, 40 cycles of 95°C for 30 s, 60°C for 20 s, 72°C for 30 s, and a final elongation step of 72°C for 5 min. Samples were analyzed using a StepOnePlus Thermal Cycler (Thermo Fisher Scientific), setting the cycle threshold in the linear phase of the amplification plots.

**TABLE 1:** Primer pair sequences used in this study

	Sequence, 5′→3′	
Gene	Sense	Antisense
*Mm_Cnr2*	GGTCCTCTCAGCATTGATTTC	GCCCAGTAGGTAGTCGTTAG
*Mm_Vim*	TGAAGGAAGAGATGGCTCGT	GGAAGAAAAGGTTGGCAGAG
*Mm_Tlr2*	CTCCCACTTCAGGCTCTTTG	GCCACTCCAGGTAGGTCTTG
*Mm_Clec7a*	CAGGGAGAAATCCAGAGGAG	TAGGAAGGCAAGGCTGAGAA
*Mm_Actg1*	CCAACAGCAGACTTCCAGGATT	CTGGCAAGAAGGAGTGGTAACTG

*Actg1*, actin γ1.

### Neutrophil transcriptome analysis

Wild-type (WT) and CB_2_^−/−^ neutrophils harvested from dorsal air pouches at 6 h post–Zymosan challenge were negatively selected (Miltenyi Biotec, Bergisch Gladbach, Germany), and transcriptome analysis was carried out using the nCounter Mouse Inflammation V2 panel (NanoString Technologies, Seattle, WA, USA) consisting of 248 genes and 14 positive/negative probes. Cells (5 × 10^6^/ml) were lysed and processed according to the manufacturer’s guidelines. Data were analyzed in R (v.3.3.1) using the NanoStringDiff package (v.1.2.0) (27) and its default settings. Briefly, raw nCounter data were converted into a NanoStringSet object including 6 positive controls, 8 negative controls, and 5 housekeeping genes (*Cltc*, *Gapdh*, *Gusb*, *Pgk1*, and *Tubb5*) per sample. The data were normalized and analyzed for differentially expressed genes according to NanoStringDiff instructions following a 2-group comparison approach.

### Murine neutrophil static adhesion assay

Purified bone marrow WT and CB_2_^−/−^ neutrophils (10^5^ cells) were treated with vehicle, complement component 5a (C5a) (10 nM; R&D Systems), or N-formylmethionine-leucyl-phenylalanine (fMLP) (20 nM; R&D Systems) for 15 min at 37°C and plated in a 96-well plate precoated with 12.5 μg/ml ICAM-1–Fc [1 h at 37°C, washed in PBS and blocked in PBS with 1% bovine serum albumin (BSA) for 45 min; R&D Systems] for another incubation of 15 min at 37°C. Cells were washed in PBS, and their numbers were determined after a 10-min incubation with PrestoBlue (Thermo Fisher Scientific) at 540 nm excitation and 590 nm emission using a Pherastar plate reader.

### Human neutrophil transendothelial migration assay

Primary human dermal blood endothelial cells (HDBECs) were purchased from PromoCell and cultured in the manufacturer’s recommended endothelial cell growth medium MV (PromoCell, Heidelberg, Germany). HDBECs were seeded onto 24-well tissue culture plates after 4 passages at a seeding density yielding confluent monolayers. Prior to the adhesion assay, HDBEC monolayers were washed in endothelial cell growth medium MV warmed to 37°C and cytokine simulated using TNF-α (100 U/ml; MilliporeSigma) for 4 h at 37°C. Neutrophils were isolated from whole human blood as previously described in Cooper *et al.* (28). Neutrophils were treated with vehicle (1% DMSO), 1 μM JWH133 {a CB_2_-selective agonist; (6aR,10aR)-3-(1,1-dimethylbutyl)-6a,7,10,10a-tetrahydro-6,6,9-trimethyl-6H-dibenzo[b,d]pyran}, or 1 μM JWH133 and 1 μM SR144528 {a CB_2_-selective antagonist; 5-(4-chloro-3-methylphenyl)-1-[(4-methylphenyl)methyl]-N-[(1S,2S,4R)-1,3,3-trimethylbicyclo[2.2.1]hept-2-yl]-1H-pyrazole-3-carboxamide} for 15 min prior to use.

Prior to beginning the assay, HDBEC monolayers were washed with 37°C medium 199 (Thermo Fisher Scientific) supplemented with 0.15% w/v BSA (MilliporeSigma) to remove any residual cytokines. Treated neutrophils (0.15 × 10^6^) were cocultured with cytokine-stimulated HDBEC monolayers at 37°C for 6 min and 30 s. To remove any cells adherent by electrostatic interactions, the monolayers were washed twice with 37°C medium 199 supplemented with 0.15% w/v BSA. HDBEC monolayers and adherent neutrophils were then fixed in 2% glutaraldehyde (MilliporeSigma) for 15 min and washed twice in PBS. The extent of neutrophil adhesion and transmigration was imaged using phase-contrast microscopy with an inverted bright-field microscope (IX71; Olympus, Tokyo, Japan) at ×32 magnification. A total of 5 images of 5 different views were taken per well and processed offline using Image Pro 7 software (Media Cybernetics, Rockville, MD, USA). Neutrophils were manually tagged as being surface adherent (phase bright and rounded) or as having transmigrated (phase dark with altered morphology). Total neutrophil adhesion and mean percentage transmigration were calculated for each well.

### Data and statistical analysis

All data are reported as the mean + or ± sem of independent experiments and were analyzed using Prism v.7 (GraphPad Software, La Jolla, CA, USA). For 2-group comparisons, a Student’s *t* test was applied, whereas for multiple comparisons with 1 or 2 variables, a 1-way ANOVA with Dunnett’s multiple comparisons correction and a 2-way ANOVA with Sidak’s multiple comparisons correction were applied, respectively. Results were considered statistically significant when *P* < 0.05.

## RESULTS

### Characterization of the leukocyte recruitment pattern in CB_2_^−/−^ mice

To examine how CB_2_ regulates immune cell trafficking *in vivo*, we decided to use the dorsal air pouch model of inflammation, as it offers the advantage of an accessible administration site for inflammatory stimuli and simple quantitative collection of the inflammatory exudate. Zymosan has been used to elicit an inflammatory response in several animal models, including the dorsal air pouch (29, 30) and was therefore chosen as the inflammatory insult.

We began by analyzing the immune cell composition of dorsal air pouches from WT and CB_2_^−/−^ female mice under baseline conditions and upon challenge with Zymosan (100 μg) across a range of time points. Leukocyte numbers (total CD45^+^ cells) in the pouches were similar between WT and CB_2_^−/−^ mice under basal conditions (Supplemental Fig. S1*A*, *E*, *I*). In addition, we found that neutrophils [CD45^+^, CD115^−^, Ly-6G^+^, and Ly-6C^lo^; Supplemental Fig. S1*C*, *G*] as well as the Ly-6C^lo^ and Ly-6C^hi^ monocyte (CD45^+^, CD115^+^; Supplemental Fig. S1*D*, *H*) populations were present in the pouches of both WT and CB_2_^−/−^ animals, and their numbers were comparable between the 2 genotypes (Supplemental Fig. S1*I–L*).

**Figure 1:**
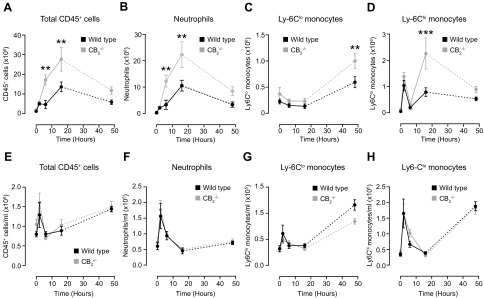
CB_2_ deficiency results in an exaggerated acute inflammatory response in the dorsal air pouch inflammation model. Dorsal air pouches of 8–10-wk-old female WT and CB_2_^−/−^ mice were injected with 100 μg Zymosan and lavaged at the indicated time points. *A–D*) Absolute numbers of total CD45^+^ cells (*A*), neutrophils (CD45^+^, Cd115^−^, Ly-6G^+^, Ly-6C^lo^) (*B*), Ly-6C^lo^ (*C*), and Ly-6C^hi^ monocytes (CD45^+^, CD115^+^) (*D*) were determined by flow cytometry. *E–H*) Blood was also withdrawn from the same animals and numbers of total CD45^+^ cells (*E*), neutrophils (*F*), Ly-6C^lo^ (*G*), and Ly-6C^hi^ (*H*) monocytes per milliliter of blood were determined by flow cytometry. Data are means ± sem (*n* = 5–11 animals/group). Statistical analysis was conducted by 2-way ANOVA with Sidak’s multiple comparisons correction. **P < 0.01, ***P < 0.001.

Zymosan injection into pouches of WT animals led to an increase in the numbers of total CD45^+^ cells, which reached a peak at 16 h and returned to baseline at 48 h. CB_2_-deficient animals demonstrated a significantly higher (*P* < 0.01) 4-fold influx of total CD45^+^ cells and neutrophils at 6 and 16 h (**Fig. 1*A, B***). Analysis of CD45^+^ cell composition revealed that neutrophils were the dominant population, making up 60–70% and 75–80% of all leukocytes at 6 and 16 h, respectively.

In contrast, Ly-6C^lo^ monocyte numbers remained relatively constant in both genotypes until 48 h, when a significantly higher (*P* < 0.01) influx was seen in mice lacking CB_2_ (Fig. 1*C*). Conversely, the number of Ly-6C^hi^ monocytes rose sharply at 2 h and almost returned to baseline at 6 h in both WT and CB_2_-deficient animals (Fig. 1*D*). There was a significantly (*P* < 0.001) more potent secondary mobilization of Ly-6C^hi^ monocytes in CB_2_^−/−^ animals at 16 h compared with WT controls (Fig. 1*D*).

Taken together, our data show that genetic deletion of CB_2_ results in augmented acute recruitment of CD45^+^ cells and, particularly, neutrophils and Ly-6C^hi^ monocytes to the site of inflammation in the dorsal air pouch model. Of note, the kinetics of Ly-6C^hi^ monocyte recruitment differ from those of neutrophils in that their trafficking is exacerbated during a second wave of leukocyte recruitment.

### Increased leukocyte recruitment to the air pouch in CB_2_-deficient mice is not due to increased blood leukocyte numbers

To assess whether the observed increased neutrophil and Ly-6C^hi^ monocyte recruitment to the pouches of CB_2_^−/−^ animals was due to a parallel increase in their numbers in the blood, we next examined the immune cell composition in the circulation of WT and CB_2_^−/−^ mice during the inflammatory challenge. Zymosan administration caused a rapid increase in the number of total CD45^+^ cells, neutrophils, and Ly-6C^hi^ monocytes in the blood of both WT and CB_2_^−/−^ animals, which returned to baseline at 16 h (Fig. 1*E*, *F*, and *H*). Interestingly, the numbers of Ly-6C^hi^ monocytes exhibited a secondary peak at the 48-h time point (Fig. 1*H*). The numbers of Ly-6C^lo^ monocytes remained stable until 48 h, when a 3-fold increase was seen in both genotypes (Fig. 1*G*). None of the dynamic changes in cell numbers in the air pouch was associated with statistically meaningful differences in leukocyte numbers in the blood of WT and CB_2_^−/−^ animals at any time point analyzed. Collectively, our findings show that the increased recruitment of neutrophils and Ly-6C^hi^ monocytes into the air pouch of CB_2_^−/−^ animals cannot be attributed to changes in leukocyte numbers in the blood.

### Elevated levels of MMP-9 and monocyte-recruiting chemokines are observed in pouch exudates of CB_2_^−/−^ mice

**Figure 2:**
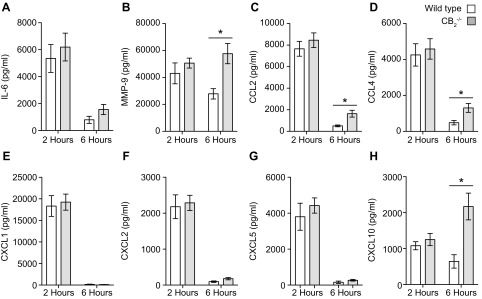
MMP-9 and CC chemokine secretion is higher in the dorsal air pouch exudates of CB_2_^−/−^ mice. Dorsal air pouches of 8–10-wk-old female WT and CB_2_^−/−^ mice were injected with 100 μg Zymosan and lavaged 2 and 6 h later for inflammatory mediator level measurement. IL-6 (*A*), MMP-9 (*B*), CCL2 (*C*), CCL4 (*D*), CXCL1 (*E*), CXCL2 (*F*), CXCL5 (*G*), and CXCL10 (*H*) levels were determined by ELISA or Luminex assays. Data are means ± sem (*n* = 6–14 animals/group). Statistical analysis was conducted by 2-way ANOVA with Sidak’s multiple comparisons correction. *P < 0.05.

A plausible mechanistic explanation for the augmented acute recruitment of innate immune cells to the pouch of CB_2_^−/−^ mice would be elevated production of local chemokines. We therefore measured the inflammatory mediator levels in the pouches of WT and CB_2_^−/−^ animals at 2 and 6 h following the Zymosan challenge because these time points preceded the increased acute neutrophil and Ly-6C^hi^ influx seen in CB_2_^−/−^ mice. We found no statistically significant difference in the levels of IL-6, MMP-9, CCL2, CCL4, CXCL1, -L2, -L5, or -L10 at 2 h (**Fig. 2**), whereas the levels of IL-6, CXCL1, -L2, and -L5 decreased similarly at 6 h in both genotypes. Nevertheless, the levels of MMP-9, CCL2, CCL4, and CXCL10 (Fig. 2*B–D*, *H*) were significantly higher (*P* < 0.05) in the exudates of CB_2_^−/−^ mice, implying that the increased neutrophil and Ly-6C^hi^ monocyte influx in CB_2_^−/−^ animals may be due in part to elevated local metalloproteinase and chemokine secretion.

### Male CB_2_^−/−^ animals also display an enhanced inflammatory phenotype

To rule out the possibility that our observations were gender-specific, we compared leukocyte recruitment and inflammatory mediator production in the pouches of male WT and CB_2_^−/−^ mice at 6 h upon Zymosan administration. Similar to our data with female animals, neutrophils constituted the dominant leukocyte population at 6 h (Supplemental Fig. S2*A*, *C*) and, together with total CD45^+^ cells, were significantly greater in number (*P* < 0.05) in the pouches of CB_2_^−/−^ male mice (Supplemental Fig. S2*E*, *F*). Of note, the effect size observed in male animals was smaller than that of females (Fig. 1*A*, *B* in comparison with Supplemental Fig. S2*E*, *F*). Lastly, Ly-6C^lo^ and Ly-6C^hi^ monocytes were also present in the pouches of male mice (Supplemental Fig. S2*B*, *D*), but we did not detect any differences in their absolute numbers between the genotypes at 6 h (Supplemental Fig. S2*G*, *H*).

Analysis of the levels of inflammatory mediators in the pouch exudates of CB_2_^−/−^ male mice at 6 h showed that IL-6 and CCL2 were significantly higher (*P* < 0.05) (Supplemental Fig. S2*I*, *J*), whereas the levels of CXCL1 were comparable between the 2 genotypes (Supplemental Fig. S2*K*). Altogether, these data demonstrate that the increase in neutrophil numbers at the peak time point of 6 h was not gender-dependent but reflected the altered biology during CB_2_ deficiency. However, in order to search for the potential mechanisms underlying enhanced neutrophil recruitment to the dorsal air pouch in CB_2_^−/−^ animals, we chose to focus on female mice because of the larger difference in neutrophil recruitment between WT and transgenic animals.

### Cultured cells from the pouch lining of WT and CB_2_^−/−^ mice secrete comparable levels of inflammatory mediators

**Figure 3:**
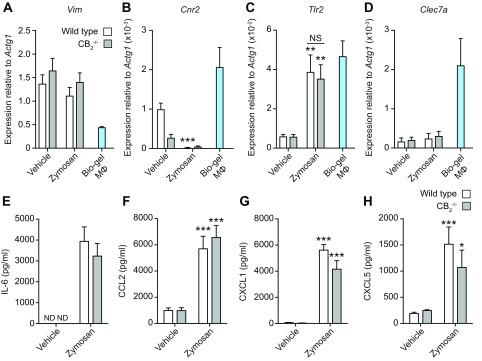
CB_2_ deletion in dorsal air pouch fibroblasts has no effect on inflammatory mediator production in response to Zymosan. *A–D*) Dorsal air pouch lining endothelial cells from WT and CB_2_^−/−^ female mice (8–10 wk old) were harvested and stimulated with 10 μg/ml Zymosan or vehicle for 6 h. *Vim* (*A*), *Cnr2* (*B*), *Tlr2* (*C*), and *Clec7a* (*D*) gene expression was determined by qPCR. *E–H*) Gene expression from Bio-gel macrophage samples is shown for comparison. IL-6 (*E*), CCL2 (*F*), CXCL1 (*G*), and CXCL5 (*H*) levels from culture supernatants were determined by ELISA. ND, not detected; NS, not significant. Data are means + sem (*n* = 5–7 biologic replicates). Statistical analysis was conducted by 2-way ANOVA with Sidak’s multiple comparisons correction. *P < 0.05, **P = 0.01, ***P < 0.001.

To identify the cell types responsible for the increased inflammatory mediator secretion observed in CB_2_^−/−^ mice, we isolated the mesothelium lining of WT and CB_2_^−/−^ pouches and tested the response to *in vitro* Zymosan stimulation. The presence of *Vim* expression in WT and CB_2_^−/−^ pouch lining cells confirmed their fibroblast-like phenotype (**Fig. 3*A***). Under basal conditions, both WT and CB_2_^−/−^ pouch fibroblasts expressed *Cnr2* (residual *Cnr2* expression was detectable in fibroblasts from CB_2_^−/−^ mice) and low levels of the Zymosan receptor Tlr2 and Dectin-1 (*Clec7a*) transcripts (Fig. 3*B*). Following stimulation with Zymosan for 6 h, the fibroblasts from both genotypes down-regulated *Cnr2* expression significantly (*P* < 0.001) and increased (*P* < 0.01) that of *Tlr2* to a similar level with that seen in Bio-gel–elicited macrophages (Fig. 3*C*). In contrast, *Clec7a* expression levels remained negligible in pouch lining cells from both genotypes, even after Zymosan stimulation (Fig. 3*D*).

Finally, Zymosan stimulation of both WT and CB_2_^−/−^ pouch fibroblasts induced a significant increase (*P* < 0.001) in the production of IL-6, CCL2, CXCL1, and CXCL5, although no statistically significant difference was observed in the levels of any of these mediators between genotypes (Fig. 3*E–H*). Our results demonstrate that the pouch lining contributes to the inflammatory response to Zymosan challenge, but the absence of endogenous CB_2_^−/−^ does not enhance the production of common neutrophil and Ly-6C^hi^ monocyte–recruiting inflammatory mediators. It is therefore unlikely that these fibroblast-like cells are responsible for the increased innate immune cell recruitment seen in CB_2_^−/−^ mice.

### Absence of CB_2_ on neutrophils results in enhanced recruitment to local site of inflammation

To test the hypothesis that the specific absence of CB_2_ on neutrophils may directly alter their migratory behavior, we generated bone marrow chimeric animals following the scheme detailed in Supplemental Fig. S3*A*. To confirm success of the bone marrow repopulation, tail blood was withdrawn from each animal 5 wk later to assess the relative proportion of CD45.1 to CD45.2 cells (Supplemental Fig. S3*B*, *C*). Quantification of cell numbers clearly demonstrated that there was no difference in the absolute levels of CD45.1 and CD45.2 leukocytes in the blood of both WT and CB_2_^−/−^ recipient animals (Supplemental Fig. S3*D*).

**Figure 4:**
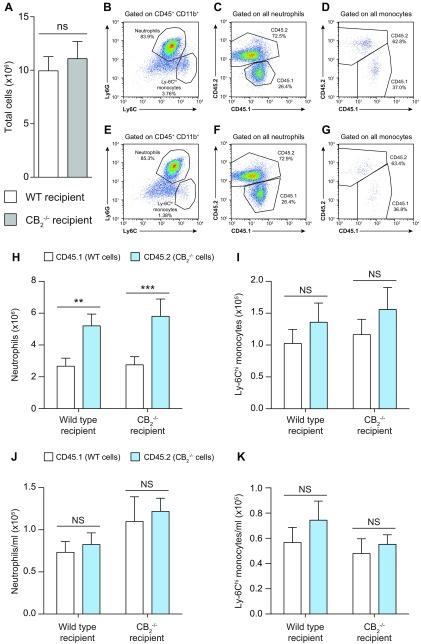
Lack of CB_2_ in hematopoietic cells is responsible for the increased neutrophil recruitment to the dorsal air pouches of CB_2_^−/−^ animals. WT and CB_2_^−/−^ bone marrow chimeric female mice (14–16 wk old) containing an equal mix of CD45.1^+^ (WT) and CD45.2^+^ (CB_2_^−/−^) myeloid cells were used in the dorsal air pouch inflammation model. *A–G*) Dorsal air pouches were lavaged 6 h after 100 μg Zymosan injection, and absolute numbers of total CD45^+^ cells (*A*) as well as representative dot plots of CD45^+^CD11b^+^ cells (*B*, *E*), neutrophils (*C*, *F*), and monocytes (*D*, *G*) were determined by flow cytometry from WT and CB_2_^−/−^ recipient animals, respectively. *H–K*) Absolute numbers of CD45.1^+^WT and CD45.2^+^CB_2_^−/−^ neutrophils (*H*) and Ly-6C^hi^ monocytes (*I*) recruited to the dorsal air pouches of WT and CB_2_^−/−^ recipient mice. Blood was also withdrawn from the same animals, and numbers of CD45.1^+^WT and CD45.2^+^CB_2_^−/−^ neutrophils (*J*) and Ly-6C^hi^ monocytes (*K*) per milliliter of blood were determined by flow cytometry. NS, not significant. Data are means + sem (*n* = 9–11 animals/group). Statistical analysis was conducted by 2-way ANOVA with Sidak’s multiple comparisons correction. **P < 0.01, ***P < 0.001.

Assessment of leukocyte recruitment to the pouches of WT and CB_2_^−/−^ recipient chimeric mice 6 h after Zymosan injection revealed no difference in the number of recruited total CD45^+^ cells (**Fig. 4*A***) between the 2 genotypes, whereas neutrophils were the most prevalent immune cell type (Fig. 4*B*, *E*). Consistent with a neutrophil-specific effect of CB_2_ deficiency during the acute phase of inflammation, our data revealed that this population was made up of proportionally more CD45.2^+^ than CD45.1^+^ cells, regardless of the recipient genotype (Fig. 4*C*, *F*). Quantification of CD45.1^+^ and CD45.2^+^ neutrophils in both WT and CB_2_^−/−^ recipient mice conclusively demonstrated that CD45.2^+^ CB_2_^−/−^ neutrophils migrated more readily into the pouch than their CD45.1^+^ WT counterparts at 6 h (Fig. 4*H*). We observed a minor Ly-6C^hi^ monocyte population in both recipient genotypes (Fig. 4*B*, *E*) that consisted of equal proportions of CD45.1^+^ and CD45.2^+^ cells (Fig. 4*D*, *G*). Quantification of cell numbers showed that this immune cell type infiltrated the dorsal air pouch of both WT and CB_2_^−/−^ recipient animals independently of their CB_2_ expression profile (Fig. 4*I*).

Finally, to exclude the existence of disproportionate numbers of CD45.2^+^ neutrophils in the circulation of WT and CB_2_^−/−^ recipient animals as an explanation for their increased numbers in the pouches, we quantified the numbers of CD45.1^+^ and CD45.2^+^ neutrophils in blood from the same animals and found that there was no significant difference (Fig. 4*J*). The same was also true for Ly-6C^hi^ monocytes (Fig. 4*K*). Taken together, our data rule out the explanation that the increased numbers of CD45.2^+^ CB_2_^−/−^ neutrophils in the pouches of bone marrow recipient mice is due to differences in their numbers in the bloodstream and clearly demonstrate that a lack of CB_2_ on the neutrophils themselves is responsible for their increased trafficking into the pouches of CB_2_^−/−^ animals during acute inflammation.

### CB_2_^−/−^ neutrophils overexpress promigration transcripts

**Figure 5:**
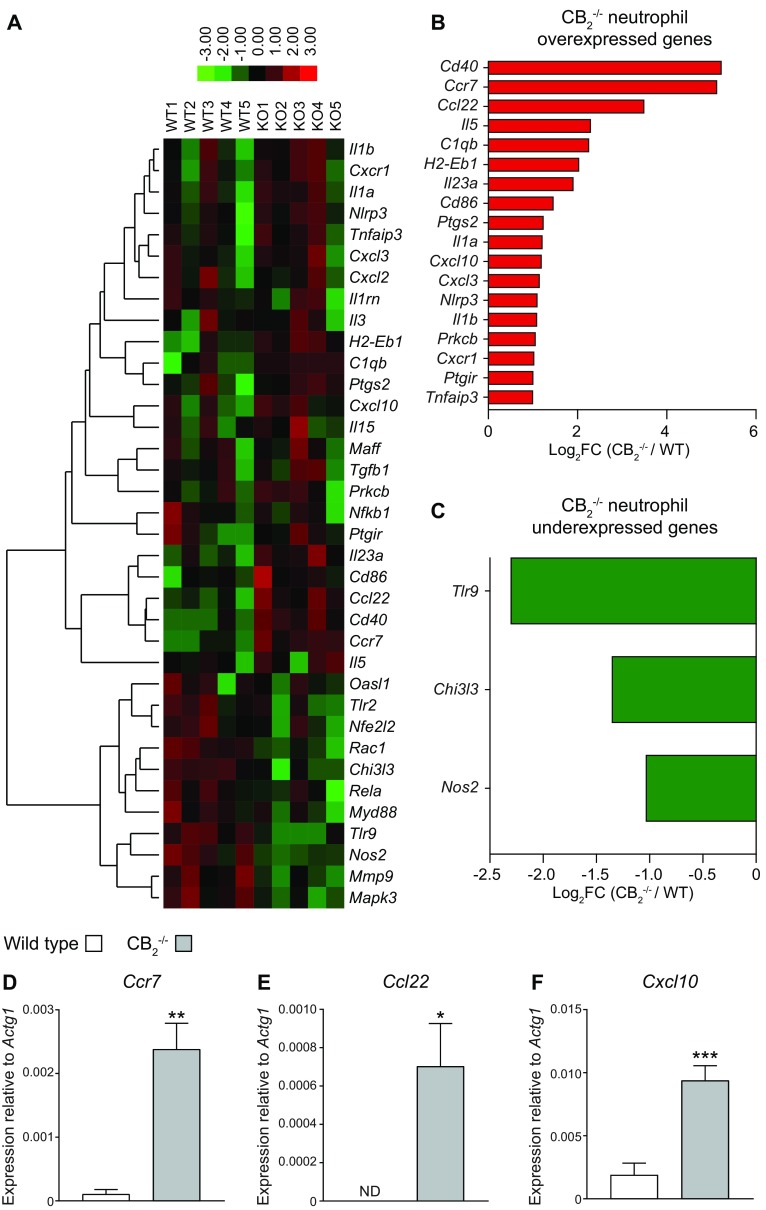
CB_2_^−/−^ neutrophils from dorsal air pouches display higher expression of migration-related molecules. Dorsal air pouches of 8–10-wk-old female WT and CB_2_^−/−^ mice were injected with 100 μg Zymosan and lavaged 6 h later. Recruited neutrophils were purified with negative selection, and their transcriptome was profiled with the nCounter Gene Expression Kit. *A*) 36 genes were differentially expressed (DE) in CB_2_^−/−^ neutrophils. Spearman correlation and average linkage were chosen as the clustering parameters in the heatmap. Each row represents 1 gene, and each column represents neutrophils isolated from an independent WT or CB_2_^−/−^ animal. *B–F*) List of at least 2-fold up-regulated (*B*) and down-regulated (*C*) DE genes in CB_2_^−/−^ neutrophils. *Ccr7* (*D*), *Ccl22* (*E*), and *Cxcl10* (*F*) gene expression was validated by qPCR. Data are means + sem (*n* = 5 independent neutrophil purifications/genotype). Statistical analysis was conducted by a 1-tailed Student’s *t* test with Welsh’s correction. *P < 0.05, **P < 0.01, ***P < 0.001,

In our experiments, neutrophils were the predominant cell type among leukocytes in the acute phase of the dorsal air pouch model. Therefore, we undertook a transcriptional analysis of neutrophils present in the air pouch exudates of WT and CB_2_^−/−^ mice. We purified neutrophils from pouches of WT and CB_2_^−/−^ animals at 6 h post–Zymosan challenge and performed a NanoString transcriptomic analysis. Comparison of the expression levels of the 243 genes present in the nCounter Mouse Inflammation panel between WT and CB_2_^−/−^ neutrophils identified 36 genes that were differentially expressed between the 2 genotypes (**Fig. 5*A***). The expression of 24 genes was significantly (*P* < 0.05) up-regulated (Fig. 5*B*), whereas 6 genes were significantly (*P* < 0.05) down-regulated (Fig. 5*C*). We additionally applied a 2-fold change cutoff to focus on differentially expressed genes with a larger effect size. Genes significantly overexpressed in CB_2_^−/−^ neutrophils belonged to several ontology categories, including chemokines and chemokine receptors (*Ccr7*, *Ccl22*, *Cxcl10*, *Cxcl3*, and C-X-C motif chemokine receptor 1), cytokines (*Il5*, *Il23a*, *Il1a*, and *Il1b*), T-cell costimulatory molecules (*Cd40*, histocompatibility 2, class II antigen Eβ, and *Cd86*), inflammasome activation [NLR family, pyrin domain containing 3 (*Nlrp3*)], complement components (complement component 1q B chain), prostaglandin pathways (post-transcriptional gene silencing 2 and prostaglandin I2 receptor), and signaling pathways (PKC-β and TNF-α–induced protein 3) (Fig. 5*B*). On the other hand, genes significantly down-regulated in CB_2_^−/−^ neutrophils were related to pathogen recognition receptors (*Tlr9*), lectins (chitinase 3-like protein 3), and nitrite production (Nos2) (Fig. 5*C*).

To validate these findings, we assessed the expression levels of representative genes related to neutrophil migration with qPCR. Expression level differences of *Ccr7* (Fig. 5*D*), *Ccl22* (Fig. 5*E*), and *Cxcl10* (Fig. 5*F*) between WT and CB_2_^−/−^ neutrophils corroborated the NanoString data. Our data demonstrate that neutrophils, which lack CB_2_, overexpress genes associated with chemotaxis and inflammatory cell recruitment during the acute inflammatory response induced by Zymosan stimulation.

### Enhanced CB_2_^−/−^ neutrophil adhesion to ICAM-1

**Figure 6:**
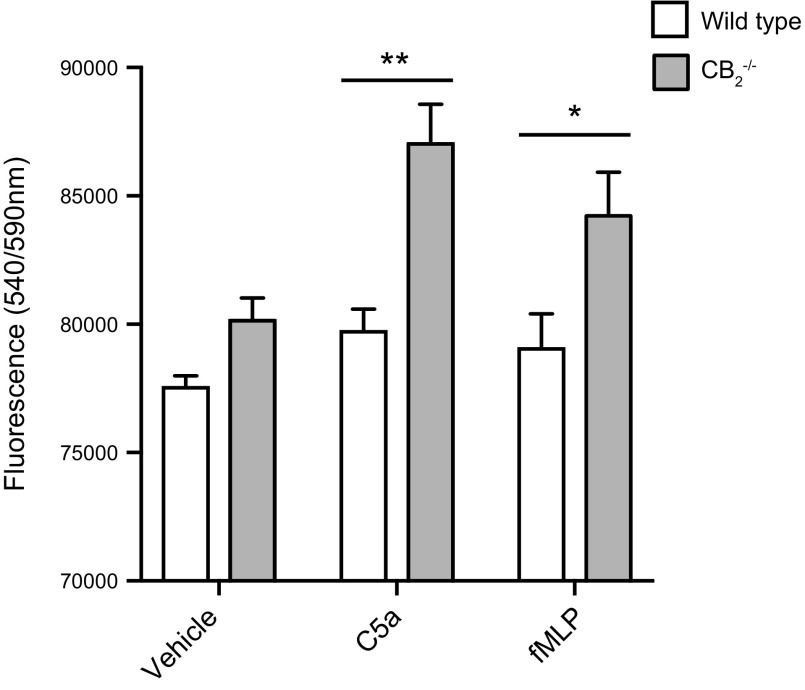
CB_2_^−/−^ neutrophils from bone marrow adhere more to ICAM-1 upon stimulation with complement and bacterial products. Bone marrows of 8–10-wk-old female WT and CB_2_^−/−^ mice were harvested, and neutrophils were purified with negative selection. Cells were incubated with vehicle, 10 nM C5a, or 20 nM fMLP for 15 min at 37°C and were then transferred to ICAM-1–precoated 96-well plates to adhere for another 15 min. Adhesion quantification was carried out with the PrestoBlue dye. Data are means + sem (*n* = 4 independent neutrophil purifications/genotype). Statistical analysis was conducted by 2-way ANOVA with Sidak’s multiple comparisons correction. *P < 0.05, **P < 0.01.

We next wanted to explore whether neutrophils lacking CB_2_ have altered adhesive properties, potentially providing a mechanistic explanation for the enhanced recruitment of CB_2_^−/−^ neutrophils in the air pouch model. Therefore, we performed an *in vitro* static adhesion assay. Briefly, purified neutrophils from the bone marrow of WT and CB_2_^−/−^ mice were stimulated with vehicle, the complement peptide C5a, or the bacterial component fMLP and then incubated in ICAM-1–treated culture plates. Following extensive washing, adhered neutrophils were quantified. Although vehicle-stimulated neutrophils from both genotypes showed no variation in adherence to ICAM-1–treated plates, CB_2_^−/−^ neutrophil numbers were significantly higher when pretreated with C5a (*P* < 0.01) and fMLP (*P* < 0.05) than their WT counterparts (**Fig. 6**). Taken together, our *in vitro* findings support a model in which the absence of CB_2_ signaling increases the adhesion of neutrophils to endothelial cell integrins, which facilitates their transmigration to sites of inflammation.

### Activation of CB2 in human neutrophils blocks endothelial transmigration

**Figure 7:**
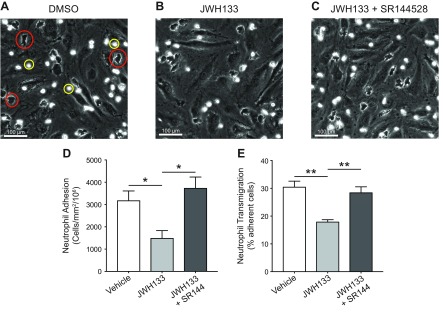
The CB_2_ agonist JWH133 blocks the endothelial adhesion and transmigration of human neutrophils in a CB_2_-dependent manner. *A–C*) Representative images of human PMNs adhered to (yellow circle) or transmigrated through (red circle) TNF-α–activated endothelial cells that were preincubated with either 1% DMSO (*A*), JWH133 (1 µM) (*B*), or JWH133 + SR144528 (both 1 µM) (*C*). *D*, *E*) Total adhesion (*D*) and transmigration (*E*) were quantified. Data are means + sem (*n* = 6 independent human donors). Statistical analysis was conducted by 1-way ANOVA with Holm-Sidak’s multiple comparisons correction. *P < 0.05, **P < 0.01.

Because a lack of CB_2_ promotes the adhesion of neutrophils to ICAM-1, and CB_2_^−/−^ mice have increased neutrophil recruitment during acute inflammation, we reasoned that pharmacological activation of CB_2_ should result in an opposing effect and would therefore block neutrophil adhesion to TNF-α–activated endothelial cells and their subsequent transmigration. To test this hypothesis, we examined human neutrophil adhesion and transmigration on a TNF-α–activated endothelial monolayer following CB_2_ agonist treatment *in vitro*. When treated with vehicle alone (1% DMSO), both adhered (phase-bright cells; **Fig. 7*A***) and transmigrated (phase-dark cells; Fig. 7*A*) neutrophils could be clearly seen. Treatment with the CB_2_-selective agonist JWH133 (1 µM) caused a significant reduction in the total number of adhered and transmigrated neutrophils (representative image, Fig. 7*B*, *D*, *E*). The reduction in both neutrophil adhesion and transmigration seen with JWH133 treatment was fully reversed by coincubation with the CB_2_-selective antagonist SR144528 (1 µM; representative image, Fig. 7*C*–*E*). We believe that taken together, these data demonstrate that the specific activation of CB_2_ in human neutrophils is sufficient to block their endothelial adhesion and transmigration.

## DISCUSSION

**Figure 8:**
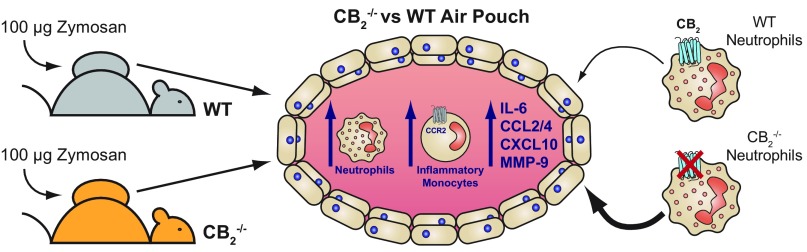
Graphical summary of the main findings presented in this study. Dorsal air pouches were established in WT and CB_2_^−/−^ mice by the dorsal subcutaneous injection of air and were subsequently injected with 100 μg Zymosan. Animals genetically deleted for CB_2_ have an exaggerated acute inflammatory response because they have significantly more neutrophils, Ly-6C^hi^ inflammatory monocytes, and proinflammatory mediators present in the pouch after Zymosan challenge in comparison with WT animals. Interestingly, it is the lack of CB_2_ on the neutrophils themselves that makes them more likely to traffic into the dorsal air pouch, likely due to CB_2_^−/−^ neutrophils having an enhanced migratory transcriptional profile and increased endothelial adhesion and transmigration.

In this study, we have demonstrated for the first time that global CB_2_ deficiency results in enhanced neutrophil and Ly-6C^hi^ monocyte recruitment in the dorsal air pouch model of inflammation, and our main findings are summarized in **Fig. 8**. The neutrophil mixed bone marrow chimera experiments unambiguously demonstrate cell-autonomous effects of CB_2_ genetic deletion on mobilized neutrophils, which exhibit an enhanced migratory transcriptional profile. Our novel findings with murine neutrophils are supported by experiments in which pharmacological activation of CB_2_ receptors on human polymorphonuclear neutrophils (PMNs) blocks endothelial transmigration *in vitro*. Previous studies have documented anti-inflammatory effects of CB_2_ agonists in a range of murine models of inflammation at single time points or single doses (15, 18, 22, 31–36). By using multicolor flow cytometry to accurately identify immune cell subsets (37, 38) and by performing a full kinetic analysis, rather than the single endpoint approaches, we were able to fully examine the role of CB_2_ throughout the acute inflammatory response. Our detailed analysis of acute inflammation in global CB_2_-knockout animals is consistent with cannabinoid signaling providing a tonic anti-inflammatory arm in the host response to injury and infection (10, 39, 40).

Importantly, the significant increase of neutrophil numbers in the pouches of CB_2_^−/−^ animals cannot be attributed to differences in their numbers in the pouch or blood under basal conditions. Hence, increased pouch neutrophil numbers must be a result of augmented mobilization to the injury site. Previous *in vitro* experiments have demonstrated that CB_2_ activation may suppress neutrophil and monocyte chemotaxis (41–43); however, additional lines of evidence in dendritic cell biology have demonstrated that CB_2_ does not inhibit chemotaxis *per se*, but rather down-regulates MMP-9 levels to reduce cell invasiveness (44). Our findings support the idea that CB_2_ regulates neutrophil invasion, potentially *via* the metalloproteinase MMP-9, because we found that a lack of CB_2_ in murine neutrophils increased their adhesion to ICAM, the specific pharmacological activation of human neutrophils blocked their endothelial transmigration, and CB_2_^−/−^ animals had significantly higher levels of MMP-9 in the pouch exudate 6 h after Zymosan challenge. Indeed, our laboratory recently demonstrated that neutrophil infiltration to the spleen is regulated by CB_2_ *via* MMP-9 reduction in a low dose endotoxemia model (45), and others have found higher MMP-9 levels in atherosclerotic lesions from CB_2_^−/−^ animals (46). Because neutrophils are a major source of MMP-9 (47), CB_2_^−/−^ neutrophils may express higher levels of MMP-9, which could enhance their invasive capacity, resulting in increased pouch neutrophil recruitment. However, it is possible that the increased levels of MMP-9 may be reflective of the increased numbers of neutrophils seen in CB_2_^−/−^ animals, and that the mechanism underlying enhanced recruitment lies elsewhere.

It has been proposed that CB_2_ mediates its antimigratory effects on leukocytes indirectly *via* the endothelium (48, 49). However, our work with mixed bone marrow chimeras and the *ex vivo* culture of pouch lining cells ruled out the involvement of stromal cells in the *in vivo* phenotype of CB_2_-deficient animals, and our transcriptomic analysis and the adhesion assay outlined in Fig. 6 identified a dysregulated phenotype of CB_2_^−/−^ neutrophils harvested directly from the site of inflammation. The up-regulated expression levels of cytokines, chemokines, chemokine receptors, complement receptors, and activation markers in CB_2_^−/−^ neutrophils further support the idea that unregulated neutrophil activation could lead to increased PMN recruitment *via* an autocrine feedback loop (50).

The second main finding of the current study is the effect of CB_2_ deficiency on Ly-6C^hi^ monocyte recruitment to the pouches. It is noteworthy that Ly-6C^hi^ monocyte trafficking follows a biphasic pattern in both genotypes and is exacerbated in CB_2_^−/−^ mice in the second wave of inflammation. In fact, Newson *et al.* (51) recently proposed that the second wave of monocytes aims to induce regulatory T-cell differentiation and resolution of the undergoing local inflammation. In our study, this would mean that CB_2_ deficiency accelerates the resolution of inflammation in the pouch. Support for this hypothesis is provided by the fact that in CB_2_^−/−^ mice, the rate at which inflammatory cell numbers returned to baseline following their peak was greater than that of WT mice. Additionally, there were significantly more pouch Ly-6C^lo^ monocytes in CB_2_^−/−^ animals 48 h after the Zymosan challenge, and these cells have been suggested to have a protective or anti-inflammatory role and to give rise to alternatively activated macrophages that aid tissue repair and the resolution of inflammation (52–54). However, further experiments will clearly be needed to confirm this hypothesis.

An obvious explanation for the increased Ly-6C^hi^ monocyte recruitment observed in CB_2_^−/−^ animals is the elevated CCL2, CCL4, and CXCL10 secretion in the exudates. It is not clear from our experiments whether these mediators are released from stromal cells at equal levels by the 2 genotypes or whether the accumulation of neutrophils in CB_2_^−/−^ mice is the main reason for their elevated levels in the air pouch. In keeping with the latter scenario, we found that CB_2_^−/−^ neutrophils overexpress *Cxcl10*, the up-regulation of which is critical for T helper cell differentiation and trafficking (55).

An important implication from this study is that endocannabinoids, signaling *via* CB_2_, act to reduce neutrophil recruitment during acute inflammation. Indeed, the endocannabinoids 2-arachidonoylglycerol (2-AG) and anandamide have been previously demonstrated to inhibit fMLP-induced human neutrophil chemotaxis and T-cell migration toward CXCL12 (56, 57). However, and seemingly at odds with this inhibitory function, multiple studies have found that 2-AG on its own can stimulate the directed migration of a range of immune cell types (58). Nevertheless, we recently demonstrated that 2-AG did not act as a chemoattractant for primary murine macrophages (26), and Oka *et al.* (59) found that 2-AG was unable to elicit chemotaxis of human neutrophils, thereby strongly suggesting that endocannabinoid-mediated immune cell migration is cell type–specific and likely not of relevance to neutrophils. Instead, we believe the findings presented here using CB_2_^−/−^ animals fit with the hypothesis that endocannabinoids acting *via* CB_2_, lead to a reduction in the inflammatory and migratory behavior of neutrophils, which limits their recruitment to sites of inflammation.

Collectively, this is in line with the vast array of studies showing that the activation of CB_2_ by its endogenous ligands results in anti-inflammatory effects (60, 61). However, the role CB_2_ plays during an inflammatory response is not so clear-cut because there is a growing body of evidence that demonstrates a proinflammatory role for the endocannabinoids both *in vitro* and *in vivo* (61). Additionally, it has been previously shown that 2-AG can actually enhance immune cell adhesion, either by acting directly on the immune cells, *per se*, or by up-regulating adhesion molecules on endothelial cells (62, 63). With regard to this latter discrepancy, we believe that cell type differences are likely responsible because neither of the aforementioned studies examined neutrophils and we have already ruled out an endothelial component to our results, as previously detailed. Why some *in vivo* studies demonstrate that endocannabinoids have proinflammatory properties remains more difficult to answer. In these situations, context is key because each inflammatory model used has its own unique pathophysiology, local context, and immune cell involvement, within which the types and levels of the endocannabinoids and receptors present may vary dramatically (64). Together, these factors likely determine how the endocannabinoid system as a whole impacts the inflammatory response, but clearly further work is needed to fully unravel the complexities of this lipid signaling system *in vivo*.

Our working hypothesis is that lack of CB_2_ on neutrophils during inflammation releases the brake in their migratory capacity. CB_2_ deficiency therefore grants these cells increased invasive capacity and activation potential, which can be detrimental *via* continued recruitment of leukocytes to the inflamed site. In summary, this study supports the notion that pharmacological activation of CB_2_ could be a suitable therapeutic avenue for the treatment of human inflammatory diseases because of its inhibitory effects on neutrophil recruitment during an acute inflammatory response.

## Supplementary Material

This article includes supplemental data. Please visit *http://www.fasebj.org* to obtain this information.

## Abbreviations

2-AG: 2-arachidonoylglycerol
BSA: bovine serum albumin
C5a: complement component 5a
CB: cannabinoid receptor
CCL: C-C motif chemokine ligand
*Ccr7*: C-C chemokine receptor type 7
*Clec7a*: C-type lectin domain containing 7A
*Cnr2*: cannabinoid receptor 2
CXCL: C-X-C motif chemokine ligand
FACS: fluorescence-activated cell sorting
FCS: fetal calf serum
fMLP: *N*-formylmethionyl-leucyl-phenylalanine
HDBEC: human dermal blood endothelial cell
ICAM: intercellular adhesion molecule
JWH133: (6aR,10aR)-3-(1,1-dimethylbutyl)-6a,7,10,10a-tetrahydro-6,6,9-trimethyl-6H-dibenzo[b,d]pyran
Ly-6C: lymphocyte antigen 6 complex, locus C
Ly-6G: lymphocyte antigen 6 complex, locus G
Ly-6C^hi^: high Ly-6C
Ly-6C^lo^: low Ly-6C
MMP-9: matrix metalloproteinase 9
PMN: polymorphonuclear neutrophil
qPCR: quantitative PCR
SR144528: 5-(4-chloro-3-methylphenyl)-1-[(4-methylphenyl)methyl]-N-[(1S,2S,4R)-1,3,3-trimethylbicyclo[2.2.1]hept-2-yl]-1H-pyrazole-3-carboxamide
*Vim*: vimentin
WT: wild type
