# Supplementary material for: Cannabinoid receptor 2 deficiency exacerbates inflammation and neutrophil recruitment
Source: FASEB J. 2019 Feb 25;33(5):6154–67. doi: 10.1096/fj.201802524R (PMC6629158; doi:10.1096/fj.201802524R)
Supplement: Supplementary file 1 [file fj.201802524R.sf1.docx]

**Supplementary Figure 1. Flow cytometry gating strategy for cell identification in the dorsal air pouch inflammation model.** Dorsal air pouches from WT and CB_2_^-/-^ mice (8-10 weeks old) were lavaged and representative dot plots of (A) total CD45^+^ cells, (B) CD45^+^CD115^-^ and CD45^+^CD115^+^ cells, (C) neutrophils (CD45^+^ CD115^-^ Ly-6G^+^ Ly-6C^lo^) and (D) Ly-6C^lo^ and Ly-6C^hi^ monocytes (CD45^+^ CD115^+^) from one WT mouse were determined by flow cytometry. Representative dot plots displaying (E) total CD45^+^ cells, (F) CD45^+^ CD115^-^ and CD45^+^ CD115^+^ cells, (G) neutrophils (CD45^+^ CD115^-^ Ly-6G^+^ Ly-6C^lo^) and (H) Ly-6C^lo^ and Ly-6C^hi^ monocytes (CD45^+^ CD115^+^) from one CB_2_^-/-^ mouse. Total numbers of: (I) CD45^+^ cells, (J) Neutrophils, (K) Ly-6C^lo^ monocytes and (L) Ly-6C^hi^ monocytes present in the dorsal air pouches of WT (white bars) and CB_2_^-/-^ (grey bars) animals under basal conditions. Data are mean + SEM, n=5-11 animals per group. Statistical analysis was conducted by a two-tailed students t-test, ns p>0.05.

**Figure S2. Male CB_2_^-/-^ animals have an exaggerated acute inflammatory response 6 hours after pouch zymosan injection.** The dorsal air pouches of male WT or CB_2_^-/-^ mice were injected with 100 µg zymosan and 6 hours later the pouches lavaged and total cell counts and populations present determined by flow cytometry. Representative dot plots of (A) neutrophils and (B) Ly6C^lo^ and Ly6C^hi^ monocytes in the dorsal air pouch of male WT mice 6 hours after zymosan injection. Representative dot plots of (C) neutrophils and (D) Ly6C^lo^ and Ly6C^hi^ monocytes in the dorsal air pouch of male CB_2_^-/-^ mice 6 hours after zymosan injection. Quantification of the number of (E) CD45^+^ cells, (F) neutrophils, (G) Ly6C^lo^ or (H) Ly6C^hi^ monocytes recruited to the dorsal air pouch 6 hours after zymosan injection in male WT (white bars) and CB_2_^-/-^ (grey bars) animals. The levels of pro-inflammatory mediators present in the air pouch were determined by ELISA and male CB_2_^-/-^ mice had significantly higher levels of (I) IL-6 and (J) CCL2 compared to WT animals. (K) There was no difference in the level of CXCL1 between the genotypes. Data are mean+ SEM, n = 6-8 independent animals per group. Statistical analysis was conducted using the unpaired student's t-test. ns P > 0.05, * P < 0.05, ** P < 0.01.

**Supplementary Figure 3. Generation of mixed bone marrow chimeric animals.** (A) Scheme detailing the strategy used to generate mixed bone marrow chimeric animals. Bone marrow cells were isolated from 8-10 week old female B6.SJL animals expressing the CD45.1 and 8-10 week old female B6.129 CB_2_^-/-^ animals expressing the CD45.2 surface antigen, were mixed in equal amounts and were injected intravenously into female C57BL/6 WT and CB_2_^-/-^ animals (8-10 weeks old) which had been previously irradiated with 5 Gy twice to deplete their endogenous bone marrow. The mice were allowed to reconstitute their bone marrow and 4 weeks later, blood was withdrawn from the tail vein and the proportion of CD45.1^+^/CD45.2^+^ cells was determined by flow cytometry as shown in representative dot plots from one WT (B) and one CB_2_^-/-^ (C) recipient mouse and (D) the absolute numbers of CD45.1^+^ and CD45.2^+^ cells. Data are mean+SEM, n=9-11 animals per group. Statistical analysis was conducted by two-way ANOVA with Sidak's multiple comparisons correction, ns p>0.05.
